# MAPKAPK2: the master regulator of RNA-binding proteins modulates transcript stability and tumor progression

**DOI:** 10.1186/s13046-019-1115-1

**Published:** 2019-03-08

**Authors:** Sourabh Soni, Prince Anand, Yogendra S. Padwad

**Affiliations:** 1 Pharmacology and Toxicology Laboratory, Food and Nutraceuticals Division, CSIR-Institute of Himalayan Bioresource Technology (CSIR-IHBT), Palampur, Himachal Pradesh India; 2 Academy of Scientific and Innovative Research, Chennai, Tamil Nadu India

**Keywords:** Mitogen-activated protein kinase-activated protein kinase 2 (MK2), RNA binding proteins (RBPs), Adenine/uridine-rich elements (AREs), 3′-untranslated region (3′-UTR), Transcript stability, Inhibitors, Therapeutics

## Abstract

The p38 mitogen-activated protein kinase (p38MAPK) pathway has been implicated in a variety of pathological conditions including inflammation and metastasis. Post-transcriptional regulation of genes harboring adenine/uridine-rich elements (AREs) in their 3′-untranslated region (3′-UTR) is controlled by MAPK-activated protein kinase 2 (MAPKAPK2 or MK2), a downstream substrate of the p38MAPK. In response to diverse extracellular stimuli, MK2 influences crucial signaling events, regulates inflammatory cytokines, transcript stability and critical cellular processes. Expression of genes involved in these vital cellular cascades is controlled by subtle interactions in underlying molecular networks and post-transcriptional gene regulation that determines transcript fate in association with RNA-binding proteins (RBPs). Several RBPs associate with the 3′-UTRs of the target transcripts and regulate their expression via modulation of transcript stability. Although MK2 regulates important cellular phenomenon, yet its biological significance in tumor progression has not been well elucidated till date. In this review, we have highlighted in detail the importance of MK2 as the master regulator of RBPs and its role in the regulation of transcript stability, tumor progression, as well as the possibility of use of MK2 as a therapeutic target in tumor management.

## Background

A variety of stimuli evokes specific responses in cells via p38 mitogen-activated protein kinase (p38MAPK) signal pathway activation. The stress-activated p38MAPK signaling pathway regulates a plethora of cellular processes in particular apoptosis, cell division, cell invasion, and inflammatory response [1]. p38MAPK pathway’s downstream substrate, mitogen-activated protein kinase-activated protein kinase 2 (MAPKAPK2 or MK2) is involved in the post-translational regulation of cytokines as evident in MK2 knockout (MK2^−/−^) mice showing attenuated production of tumor necrosis factor (TNFα) protein when compared to wild-type mice. The mRNA levels, however, in wild-type mice were quite similar as compared to MK2^−/−^ mice, indicating regulation at the translational level which might be imparted *via* a MK2 substrate.

In response to stress stimuli, p38MAPK phosphorylates and activates MK2 which further regulates a cascade of biological events and participates in a multitude of processes like cell apoptosis [2], cell cycle [3], movement [4] and response to oxidative stress [5]. MK2 was discovered as an extracellular signal-regulated kinase (ERK1/2)-activated protein kinase that phosphorylates and inactivates heat shock protein (Hsp27) [6]. MK2 has been shown to govern the activation and deactivation of RNA-binding proteins (RBPs) [7]. These RBPs modulate the gene expression of mRNAs encoding several proto-oncogenes, cytokines, chemokines and pro-inflammatory factors that control cell-cycle progression, proliferation, angiogenesis, metastasis, and cell death [8, 9]. Experimental evidence indicates that MK2, the prime target of p38MAPK, regulates the stability of essential genes involved in tumor pathogenesis that harbour adenine/uridine-rich elements (AREs) in their 3′-untranslated region (3′-UTRs) [8].

Systemic side effects like hepatic and cardiac toxicity as well as central nervous system disorders caused by the small molecules p38MAPK inhibitors have hindered their translational use. This might be attributed to the fact that p38MAPK regulates more than sixty substrates and therefore its direct inhibitors have failed in their clinical utility due to undesired side effects [10]. This has prompted researchers to look for novel therapeutic targets in downstream regulators of this signaling pathway, prominent among them being MK2. Hence, insights into the putative role of MK2 in the post-transcriptional regulation of pathogenesis-linked transcripts have become pertinent. In this review, we have highlighted the importance of MK2 as the master regulator of RBPs and its role in the regulation of transcript stability and tumor progression. Furthermore, we have discussed the role of MK2 in various cancers and have also deliberated its significance in various cancer processes. The possibility of employing MK2 as a therapeutic inhibitor has also been reviewed.

## p38MAPK signaling pathway

**Fig. 1:**
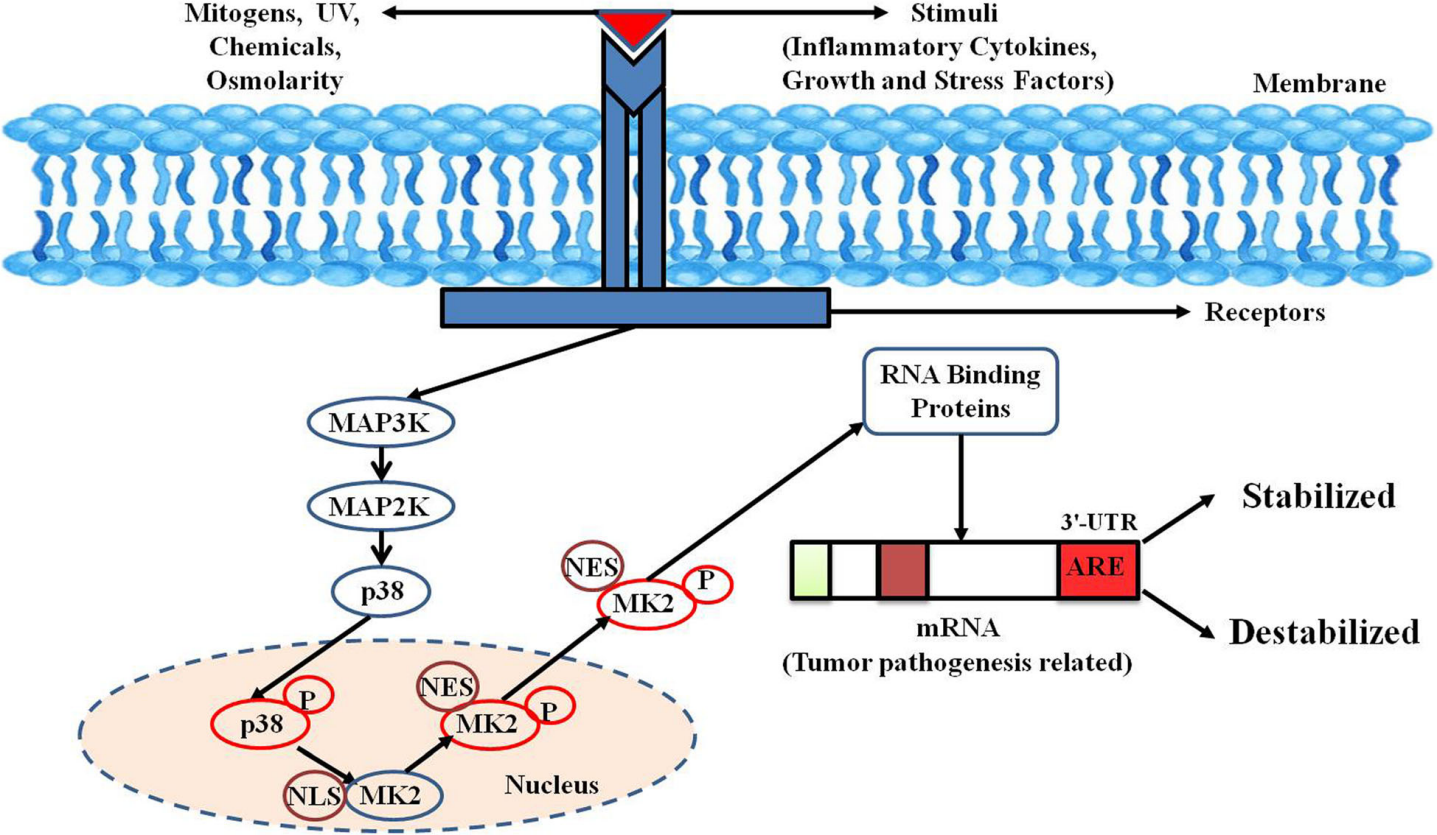
p38MAPK signalling cascade. A multitude of extracellular stimuli and mitogens lead to the activation of p38MAPK signalling pathway consisting of a kinase network as diagrammatically represented in the figure. When activated by p38, MK2 gets exported to the cytoplasm (NLS gets masked and NES is functional) where it controls the transcript stability of tumor pathogenesis related mRNAs harbouring AREs in their 3′-UTRs *via* regulation of RNA-binding proteins

p38MAPKs are key MAPKs involved in the production of important inflammatory mediators, including TNFα and cyclooxygenase-2 (COX-2). Cellular stresses/mitogens interact in a majorly receptor-mediator manner and help trigger the phosphorylation of a MAPK kinase kinase (MAP3K) specifically which further causes the phosphorylation of its downstream substrate MAPK kinase (MAP2K). After MAP2K phosphorylation, its substrate MAPK is subsequently phosphorylated (Fig. 1). Activated MAPKs further leads to the phosphorylation and activation of several downstream protein kinases, proto-oncogenes, and transcription factors [11].

### Major kinases in the p38MAPK signaling pathway

MAPK pathways comprises of an array of three kinases: Firstly, a MAP3K which is responsible to activates a MAP2K that in turn phosphorylates and activates a MAPK which occurs via a dual phosphorylation in the activation motif (Thr-X-Tyr where X could be any amino acid). Mammalian cells are known to express fourteen MAPKs which can be further segregated into groups based on sequence homology. The classical MAPKs are ERK1 and ERK2 with MAP2Ks, MKK1 or MKK2 activating them. Four isoforms of the p38MAPK family are known (p38α, p38β, p38γ, and p38δ), and these are activated by the MAP2Ks, MKK3, and MKK6 [12].

### Downstream substrates of the p38MAPK signaling pathway

There are numbers of substrates downstream of p38MAPK signaling pathways. MK2 and MK3 were the first p38MAPK substrates identified [13]. Phosphorylated MK2 or MK3 can activate a variety of substrates, such as small Hsp27 [14], cyclic AMP-responsive element-binding protein (CREB) [15], and tristetraprolin (TTP), a RBP, known to causes mRNA destabilization thus referring at p38MAPK’s role in mRNA stability [16]. It has been shown that p38MAPK modulates MK2 expression both transcriptionally and post-transcriptionally in murine cell lines and embryos while it is lost in p38^−/−^ mice [17].

## Mitogen-activated protein kinase-activated protein kinase 2

p38MAPK’s downstream substrate responsible for a plethora of signaling cascades in response to numerous extracellular stimuli ranging from apoptosis, cell division and differentiation, cell motility to inflammation is a Ser/Tyr protein kinase, MK2 [6]. MK2 acts as an important driver in the signaling pathways triggered in reply to DNA damage. A recent report has identified MK2 as protumorigenic with its role been shown in tumor progression [18]. Past reports have elucidated the expression of MK2 in a variety of cell types such as endothelial cells [19], smooth muscle cells [20], and cancers [21].

### MK2 substrates

Upon activation MK2 phosphorylates various substrates and leads to regulation of many different biological processes. The first identified MK2 substrates were Hsp25 and Hsp27 [22]. It has been reported that Hsp27 phosphorylation by MK2 causes remodeling of actin cytoskeleton which leads to cell motility [23]. MK2 increases interleukin (IL)-6 and TNF-α production by stabilizing their mRNAs or promoting its translation [24]. MK2 could phosphorylate several important cancer-related proteins, such as cell division cycle 25 (Cdc25B/C) [25], polo-like kinase 1 (Plk1) [26], tuberin (TSC2), and the ARE-binding proteins (AU-rich element RNA-binding protein 1 (AUF1), human antigen R (HuR), TTP), which are responsible in modulating transcript stability of many genes, like TNFα, Cyclin D1, Plk3, c-Fos, c-Myc, and matrix metalloproteinase (MMP) affecting cell metabolism, differentiation, and carcinogenesis [27] (Table 1). The physiological roles of these substrates are quite different, and each contains a unique and specific amino acid motif, such as X-X-Hyd-X-R-X-X-S-X-X (where Hyd is a bulky hydrophobic residue), essential for efficient MK2-mediated phosphorylation [25, 28]. Recent experimental evidence elucidated that MK2 plays an important role in the maintenance of genomic stability by contributing to the G2/M and the mitotic spindle checkpoints [7].

**Table 1:** MK2 regulates transcript stability via RBPs

RNA Binding Protein	Target mRNA	Influence on mRNA	References
AUF1	GM-CSF	Destabilized	[63]
	IL-6	Destabilized	[107–109]
	TNF-α	Destabilized	[47, 106]
	VEGF	Destabilized	[77]
HuR	COX-2	Stabilized	[90]
	Cyclins	Stabilized	[117]
	GM-CSF	Stabilized	[68]
	HIF-1α	Stabilized	[112, 113]
	IL-6	Stabilized	[120]
	IL-8	Stabilized	[120]
	MMP-9	Stabilized	[58]
	p21	Stabilized	[122]
	p27	Represses Translation	[122]
	TGF-β	Stabilized	[120]
	TNF-α	Stabilized	[120]
	VEGF	Stabilized	[113, 120]
TTP	COX-2	Destabilized	[110]
	GM-CSF	Destabilized	[68]
	IL-1	Destabilized	[107]
	IL-6	Destabilized	[107]
	IL-8	Destabilized	[107]
	MMP-9	Destabilized	[109]
	TNF-α	Destabilized	[146]
	VEGF	Destabilized	[108–110]

MK2 modulates the transcript stability and translation of various mRNAs (containing ARE in their 3′-UTRs) playing essential roles in various cellular and tumorigenic processes through RBP-mediated regulation as listed here. The list indicated in this table is not exhaustive but provides information about the important MK2-regulated transcripts. The numbers mentioned in brackets depict references for the same

### Structure and location of MK2

**Fig. 2:**
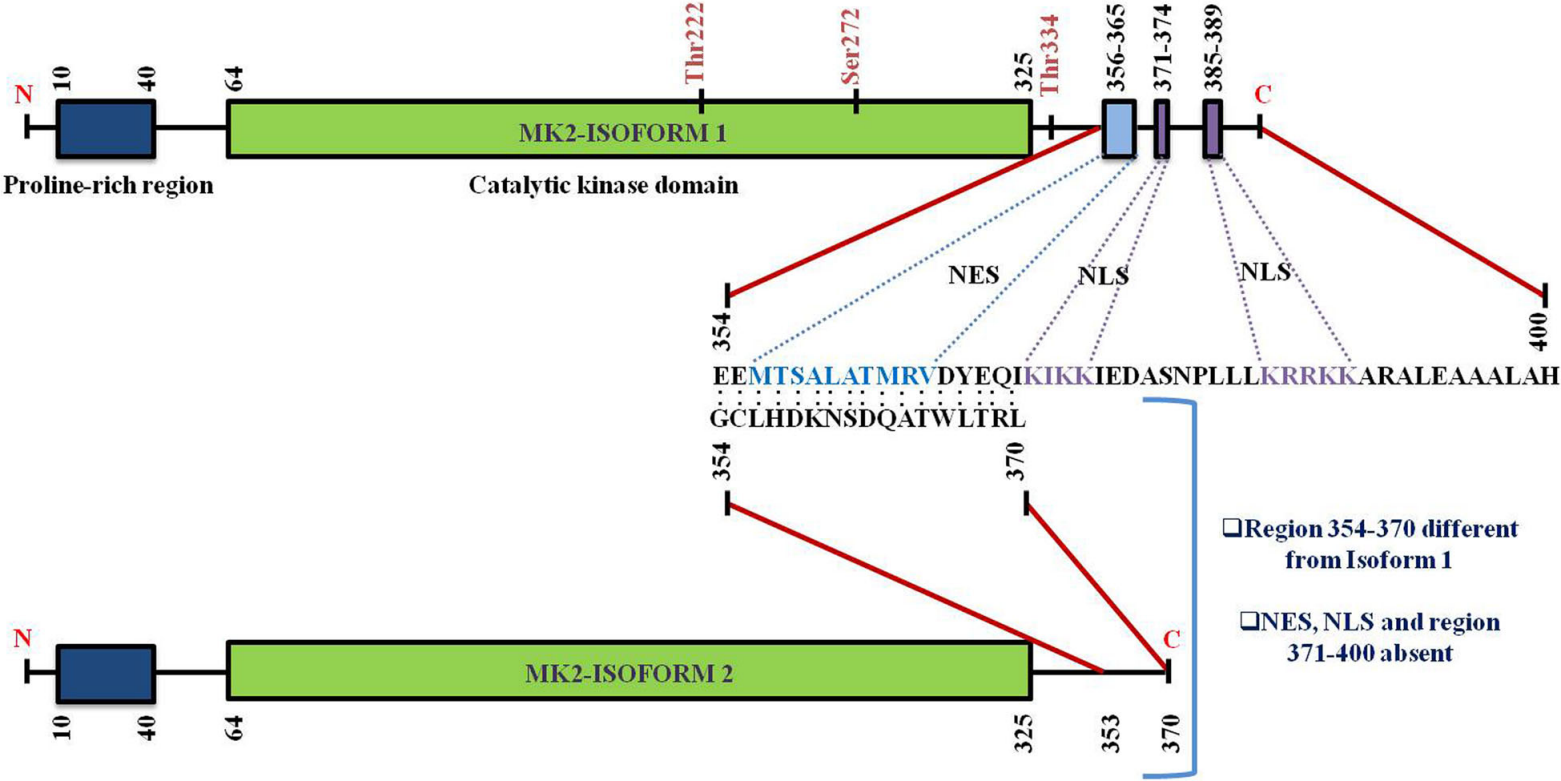
Structure of MK2 and its isoforms. Figure presents the schematic representation of M2 (Isoform 1 and 2) structure with numbers highlighting the amino-acid (a.a.) residues of various domains. N-terminal contains a proline-rich region (10–40 a.a.) followed by catalytic kinase domain (64–325 a.a.) in both the isoforms. The larger isoform (isoform 1, 400 a.a.) consists of a nuclear export signal (NES) (356–365 a.a.) and a bipartite nuclear localization signal (NLS) (371–374 and 385–389 a.a.) located at the C-terminal region. The shorter isoform (isoform 2, 370 a.a.) on the other hand does not have the NES and NLS domain. Figure also clearly indicates that the region 354–370 of isoform 2 is different from isoform 1 (sequence alignment portrayed). The three phosphorylation sites of p38 have also been marked

Human MK2, a 400-residue enzyme, contains in its N-terminus two proline-rich regions followed by the kinase and the C-terminal regulatory domain [13]. Except for MK3/4, a very low homology has been shown by the kinase domain with other serine/threonine kinases. On the other hand, no significant homology has been reported in the N-terminal proline-rich and the C-terminal regulatory domain with other non-MAPKAPK proteins. A nuclear export signal (NES) and a bipartite nuclear localization signal (NLS) are located in the C-terminal regulatory domain [29] (Fig. 2). Pull-down assays with MK2 and p38MAPK indicate that the C-terminal region 366–390 represents the p38-docking region [30]. The C-terminal regulatory domain of MK2 (also MK3) contains a functional bipartite NLS, 371–374 and 385–389, respectively which is responsible for MK2’s location predominantly in the nuclei of resting cells. Conversely, a functional NES (a motif with the sequence 356–365) which is located in the N-terminal region to the NLS is responsible for triggering nuclear export following MK2 activation [10, 30] (Fig. 2).

Before stimulation, both p38MAPK and MK2 are predominantly located in the nucleus, but they quickly translocate after stimulation to the cytoplasm together in a phosphorylation-dependent manner [29, 30]. Phosphorylation of MK2 by p38MAPK occurs in the nucleus and involves the interaction between the enzymatic and catalytic domains of p38MAPK and the NLS of MK2 [31]. Literature reports revealed that two kinase domain residues of MK2 (T222 and S272) and one residue located outside the kinase domain (T334) gets phosphorylated by p38MAPK (Fig. 1). These phosphorylations have been shown to be required for maximal activation of MK2 in vitro in mutagenesis studies [32]. MK2 activation occurs via the selective phosphorylation of T222 and T334. Phosphorylation at T334 abrogates the interaction between kinase and the C-terminal regulatory domain resulting in NES being available for binding to the nuclear receptor as revealed by the crystal structure of MK2 [33]. Once MK2 masks the NLS at the C-terminal end by phosphorylation, it is rapidly exported to the cytoplasm *via* Exportin 1-dependent mechanism to phosphorylate their downstream cytosolic targets [30] (Fig. 1).

There are many reports in the literature that confirm the role of MK2 phosphorylation at T222 located in the activation loop, S272 (catalytic domain), and T334 (outside the catalytic domain within the C-terminal region) in its activation [32]. It has been proposed that an amphiphilic α-helix motif situated within the C-terminus region of MK2 blocks the binding of MK2 with its substrates [34]. There is a hypothesis that suggests the role of T222 and T334 dual phosphorylation in repositioning this α-helix thereby resulting in an enhanced catalytic activity.

**Fig. 3:**
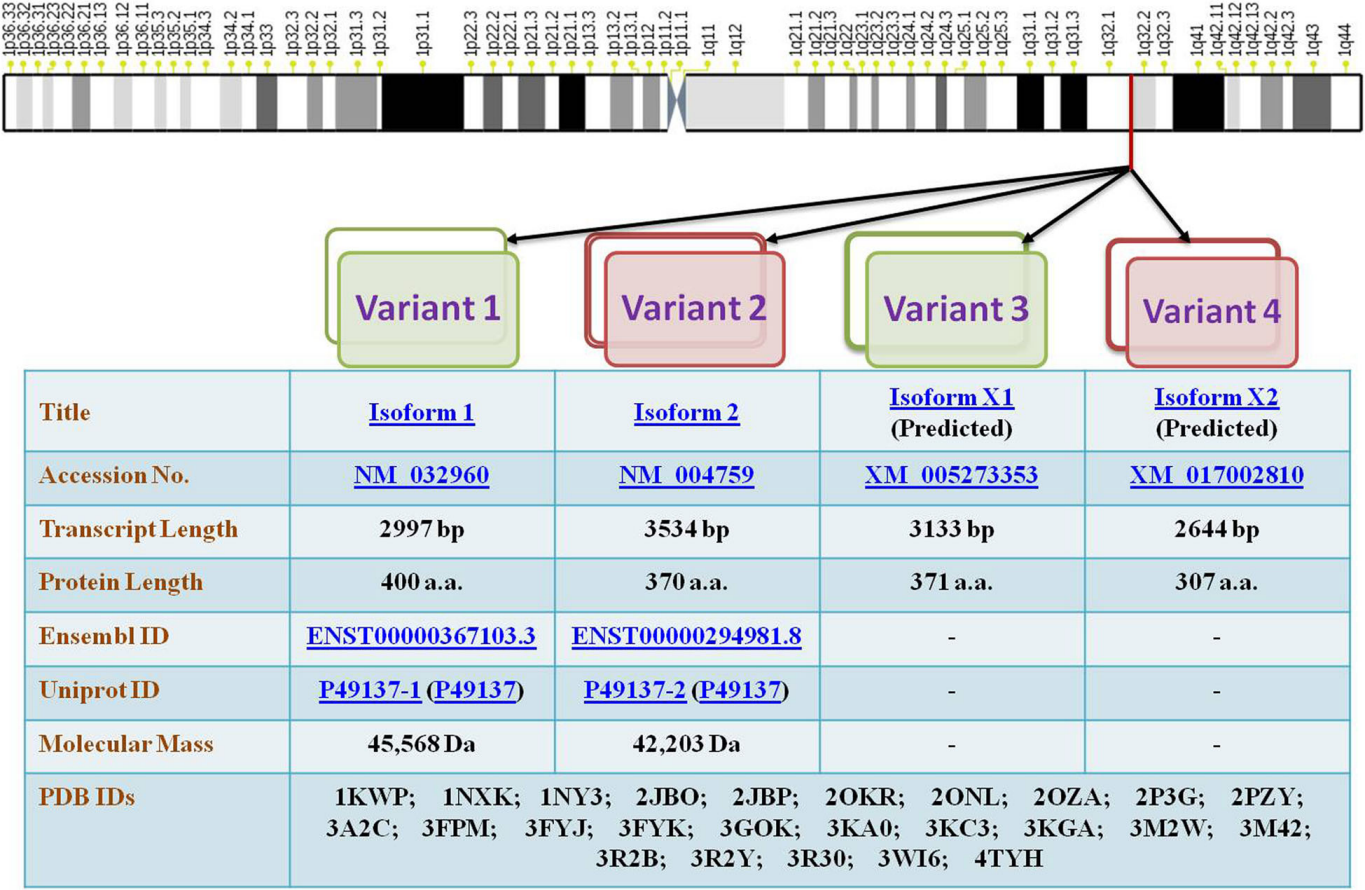
MK2 variants. Pictorial representation of the chromosomal location of MK2 and its various variants is provided in the figure. Details of all the MK2 variants discovered so far has been provided for in-depth and detailed technical information. The chromosome map is based on Ensembl’s GRCh38.p10 ideogram

MK2 has been shown to possess different splice variants and protein isoforms (Fig. 3). Sodium dodecyl sulphate-polyacrylamide gel electrophoresis (SDS-PAGE) [6] and chromatography [35] led to the description of two differentially spliced MK2 isoforms which have comparable migration intensity and which might have arisen as a result of limited proteolysis or post-translational modifications of MK2. The first variant, MK2, contains an NES, NLS and a putative p38-docking domain located near the carboxy terminus [6]. The second shorter variant of MK2 (isoform 2) [13] contains an identical N-terminal kinase domain but lacks NES, NLS and a p38-docking domain [29, 30] and bears the substitutive sequence GCLHDKNSDQATWLTRL in place of 354–400 sequence of isoform 1 [10] (Fig. 2). Recently, automated computational analysis and annotation using gene prediction method have shown that there are two more isoforms of MK2 as detailed in Fig. 3.

### MK3

The main focus of our review is MK2, but it is still important to discuss MK3 in brief [36]. This kinase has much lower expression levels as compared to MK2 [37], but possesses high structural identity and shares approximately similar substrate range with MK2 thus implying almost identical functional roles in biological systems [38]. The C-terminus of MK3 contains NLS and NES motifs rendering its unphosphorylated form located in the nucleus until p38MAPK-dependent phosphorylation induces its translocation to the cytoplasm. Additionally, MK3 could control cytokine biosynthesis in addition to MK2 due to its involvement in post-transcriptional changes in the ARE-containing mRNAs targeted by MK2 [39]. Furthermore, as compared to MK2^−/−^, the double knockout mice (MK2^−/−^/MK3^−/−^) had a higher reduction of lipopolysaccharide (LPS)-induced TNFα production [37]. Strikingly, functional dissimilarities among MK2 and MK3 have been portrayed [40].

### Copy number variations in MK2

MK2 has been reported to be oncogenic with its involvement shown in growth and invasion of tumors [5]. Hence, genetic variations in MK2 might play a role in susceptibility and prognosis of cancer. Presently, several copy number variations (CNVs) have been shown to be associated with human diseases including cancers [41, 42]. Studies in the past have reported CNVs causing MK2 overexpression to influence prognosis of tumors [43]. Similarly, CNV-30450 which duplicates the MK2 promoter was shown to increase the risk and lead to poor prognosis of lung cancer [44]. The same group further assessed the correlation of this CNV to nasopharyngeal cancer (NPC) risk [45]. Recently, it was demonstrated that there is a loss of MK2 copy number in non-small cell lung cancer (NSCLC) [46]. These studies have highlighted the need of understanding CNVs and other genomic changes in MK2 as they might act as biomarkers for assessing susceptibility, predicting risk and prognosis of cancers.

## Biological functions of MK2

**Fig. 4:**
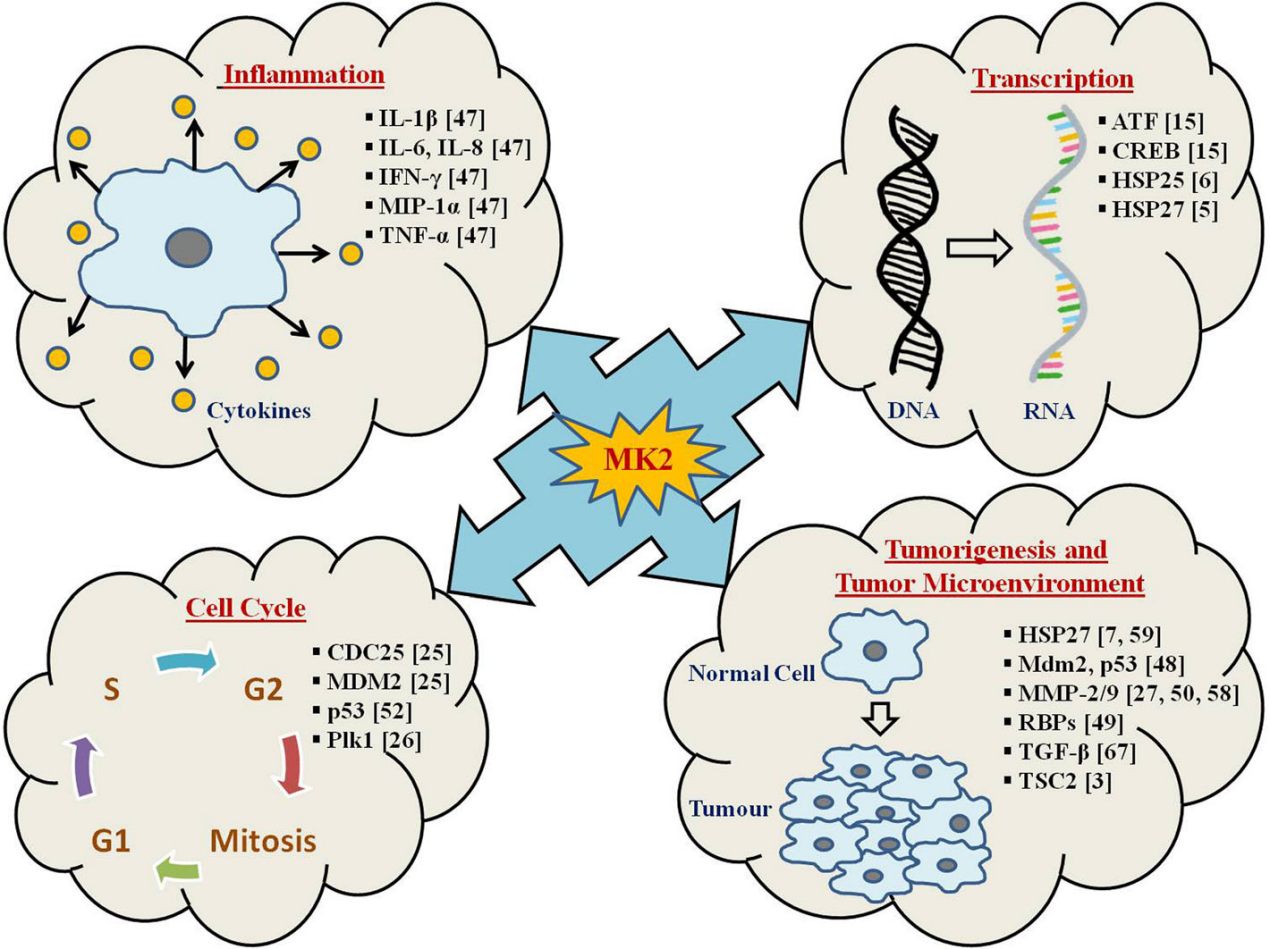
MK2 is the master regulator of tumorigenesis. The figure represents a list of MK2-regulated downstream substrates (with corresponding references highlighted in bracket) in various cellular pathways. The MK2 target genes represented here are play a vital role in cellular processes like cell cycle, inflammation, transcription, tumorigenesis and tumor microenvironment. The list is intended to be an illustration rather than being comprehensive

The biological functionalities of MK2 have not been well elucidated till date. However, MK2^−/−^ mice showcased a significant decrease in synthesis of TNFα in response to LPS [47]. in vitro studies on MK2-deficient cells have indicated a crucial role of MK2 in pro-inflammatory mediators (TNFα, IL-1β, IL-8, IL-6, and interferon-γ (IFNγ)) production [47]. MK2 was shown to be essential for up-regulation of cytokine mRNA stability and translation which is LPS-induced and hence, for stimulating cytokine biosynthesis which is integral in inflammatory responses [47]. Recently, MK2 has been reported to be intrinsic in control of cell-cycle at CDC25- and p53-dependent checkpoints [25] (Table 1, Fig. 4). DNA damage leads to inhibition of CDC25 by CHK1 and CHK2, and it has been reported that MK2 promotes G2/M checkpoint during stress response [25]. Further, MK2 was shown to phosphorylate and activate human homolog of mouse double minute 2 (HDM2), thereby causing p53 degradation suggesting the role of MK2 in dampening the p53-mediated response to DNA damage/stress [48].

MK2 orchestrates the post-transcriptional regulation of gene expression by modulating the function of RBPs [49]. It has been demonstrated that MK2 and Hsp27 both modulate cell invasion and MMP-2 activation [50]. Targeting of MK2 could be a more viable option than p38MAPK, due to potentially limited side effects, attributed to limited downstream substrates of MK2 as compared to p38MAPK. Notably, MK2^−/−^ mice are viable and have a normal phenotype [47]. Hence, much of the research has been focussed on utilizing MK2 as a molecular target for developing therapeutics for ailments like alzheimer’s, atherosclerosis, cancer, and rheumatoid arthritis (RA). MK2 modifies the function of RBPs, but MK2’s substrate spectrum is significantly limited than p38MAPK, thereby, MK2 has emerged as an attractive anti-inflammatory and anti-cancer target.

### MK2 in cell cycle regulation

Insights into the molecular mechanisms of MK2-mediated post-transcriptional regulation indicated its involvement in cell-cycle control at the CDC25- and p53-dependent checkpoints [25, 51]. Reports have shown that MK2 phosphorylates CDC25B/C at specific sites in ultraviolet (UV)-treated osteosarcoma cells and that MK2^−/−^ causes loss of G2/M checkpoint [25] (Fig. 4). Hence, MK2 can be contemplated as one of the members of the DNA-damage-checkpoint-kinase family which acts in conjunction with CHK1 and CHK2.

p53 (the tumor-suppressor protein) is also reported to be a p38MAPK cascade target. p53 has been shown to be essential for the regulation of cell-cycle at the G1/S phase and further entry into apoptosis [52]. Strikingly, p53 is a direct substrate of p38MAPK, whereas the p53-interacting ubiquitin ligase, HDM2 which is responsible for p53 degradation has been identified as one of the MK2 targets [25]. HDM2 activation occurs as a result of its phosphorylation by MK2 which further leads to increased p53 degradation, thereby, resembling the HDM2 activation by the protein kinase B (PKB)/survival kinase AKT. Hence, it has been hypothesized that MK2 inhibits p53 activity after its stimulation by p38MAPK-mediated phosphorylation, thereby, contributing to the fine regulation of DNA-damage response. Finally, the p38MAPK/MK2 pathway has been shown to activate signaling leading to G2/M checkpoint arrest and further cell survival post-DNA damage caused due to chemotherapeutics, thus responsible for resistance to treatment regimens. Mouse double minute 2 homolog (Mdm2) acts as a p53 post-transcriptional regulator, functioning by inactivating p53 through augmenting its degradation by the proteasome and repressing its transcriptional activity, thereby, down-regulating its protein levels [53]. MK2 portrays a central role in p53 post-transcriptional regulation, as it has been reported that Mdm2 phosphorylation occurs *via* MK2. Further, MK2^−/−^ cells have elevated p53 levels with reduced Mdm2 phosphorylation [48]. Reinhardt et al. [54] demonstrated that tumors lacking functional p53 can survive the effect of DNA-damage causing chemotherapeutics *via* a p38MAPK/MK2-dependent route. Meanwhile, in p53-overexpressing cells, this pathway was dispensable for survival post-DNA damage. These reports showcase that MK2 follows different mechanisms for the regulation of cell survival in response to DNA damage.

### Post-transcriptional regulation by MK2 in inflammation

Literature suggests that MK2^−/−^ mice have enhanced resistance to endotoxic shock, attributed to impairment in the inflammatory response, in addition to a decreased production of TNFα and IL-6 cytokines upon LPS-stimulation [47]. It is quite evident now that MK2 is the prime downstream substrate of p38MAPK, and this signaling cascade regulates the stability and translation of TNFα and IL-6 mRNAs through AREs involvement in the 3′-UTRs of these transcripts (Fig. 4). TTP is an RBP that has been shown to control TNFα mRNA’s stability and translation and is a direct substrate of MK2 [30]. TTP’s phosphorylation by MK2 increases its stability and binding to 14–3-3 proteins [25] and thereby stimulates TNFα expression.

### Role of MK2 in actin remodeling

Hsp27 portrays a crucial role in remodeling of actin and cell migration. In its unphosphorylated state, Hsp27 can function as an actin filament cap-binding protein, leading to inhibition of globular actin polymerization into filamentous actin (F-actin). MK2-mediated Hsp27 phosphorylation [7] blocks capping activity, thus, promoting actin polymerization and remodeling [55] (Fig. 4). Furthermore, Hsp27 phosphorylation inhibits its multimeric self-aggregation, causing loss of its chaperone activity [56]. Association of the MK2/Hsp27 relation in cell migration and remodeling of actin is also crucial for invasion and metastasis of cancer.

### Role of MK2 in tumorigenesis and tumor microenvironment

In tumor cells, the emergence of MK2 as an alternative cell-cycle checkpoint, responsible for resistance to apoptosis caused by p53 mutation, has put forward MK2 as an effective target for combination-based cancer therapies [7]. Depending on stimuli, MK2 regulates phosphorylation, mRNA stability, and expression of various proteins involved in actin remodeling [57], cell migration [58], immune responses [47], cell cycle and apoptosis [7] (Fig. 4).

#### Role in DSS-induced colitis and colorectal cancer

In colorectal carcinoma, epithelial cell proliferation and apoptosis are the key parameters contributing to tumorigenesis. As discussed earlier, one of the most important downstream mediators of MK2’s function is Hsp27, which is phosphorylated by MK2 in response to a variety of stimuli and is strongly associated with cancer progression and metastasis [59]. A recent study on intestinal epithelial cells has shown that MK2 plays a role in progression of colon cancer through downstream activation of Hsp27, which ultimately leads to angiogenesis cytokine mediation, cell proliferation, migration, and apoptosis [60]. This study also emphasized that deletion of MK2 leads to reductions of both tumor size and invasive potential in azoxymethane (AOM)/dextran sodium sulfate (DSS) induced colon cancer in mice [60]. Surprisingly, phosphorylation of Hsp27 is not influenced by MK2 deletion thus indicating that the function of the p38MAPK/MK2/Hsp27 pathway is cell and tissue dependent in colon cancer [60].

Deletion of MK2 in intestinal mesenchymal cells had the most profound effect on tumor multiplicity and size and was found associated with decreased epithelial proliferation, increased apoptosis, and decreased angiogenesis [60]. Furthermore, induction of mesenchymal cells with various MK2 and Hsp27 inducers supported an MK2-dependent functional property of this pathway. Such stimuli which are abundant in the tumor microenvironment, induce the activation of MK2 and subsequently Hsp27, resulting in the downstream production of cytokines, chemokines, and matrix metalloproteinases (MMPs), resulting in modulation of tumor microenvironment and signaling to induce cell differentiation, survival, and growth [60] (Fig. 4). Hence, it is quite evident that MK2 regulates tumor growth and progression in the intestine and could serve as a potential therapeutic target and a promising alternative to p38MAPK inhibition.

#### Role in skin cancer

Literature reports demonstrate that MK2 is required for the development of skin tumors. It regulates inflammatory response as well as maintains DNA-damaged cells survival caused by 7,12-dimethylbenz[*a*]anthracene (DMBA) during tumor initiation [61]. MK2-deficient keratinocytes were more prone to carcinogen-induced apoptosis *via* impaired Mdm2 phosphorylation and subsequently increased p53 stabilization. This suggests an inhibitory role of MK2 in apoptosis induction during tumor promotion. A crucial mediator in response to DNA damage, the p53 protein has been shown to play a pivotal role in apoptosis induction [62].

In a nutshell, MK2 works as a double-edged sword in skin carcinogenesis as it regulates pro-inflammatory cytokine expression as well as apoptosis *via* the p53-signaling pathway. It has been reported the loss of MK2 on the one hand, causes decreased inflammatory response while on the other hand it increased p53 stabilization, thereby increasing the number of DNA-damaged cells that undergo apoptosis (Fig. 4). In conclusion, MK2 inhibitors could be potential anticancer agents and be employed to inhibit the early stages during carcinoma development.

#### Role in bladder cancer

MK2 and Hsp27 lead to activation of cell invasion and MMP-2 in prostate cancer [50], with past studies showing MAPK pathways to be activated during growth phase in bladder cancer cells [63]. Further studies have reported that p38MAPK and MK2 regulate the invasion and metastasis of bladder cancer through MMP-2 and MMP-9 mRNA stability modulation [58] (Fig. 4).

Up-regulation of MMPs is one of the processes by which p38MAPK promotes cell migration and invasion in tumors. Past reports have shown high MMP-2/9 activity in HTB9 cells, while in HTB5 cells MMP-9 activity in a basal state was low. Additionally, it has been observed that p38MAPK signaling inhibition reduces MMP-2/9 activity. Hence it could be said that active p38MAPK signaling by modulation of MMP-2/9 activity may regulate the migration/invasion in bladder cancer [58]. Furthermore, the addition of MMP-2/9 antibody led to inhibition of tumor invasion, indicating that MMP expression in bladder cancers is directly responsible for it. These reports suggested that p38MAPK pathway could regulate the activity of MMP independent of tissue inhibitor of metalloproteinases (TIMP) regulation. Indeed, it has been observed that a p38MAPK inhibitor and a dominant-negative kinase-inactive mutant of MK2 led to a significant reduction in MMP-2/9 mRNA half-life. Earlier Xu and colleagues [50] had reported the role of MK2 and Hsp27 in prostate cancer cell lines invasion. Taken together, it can be concluded that the invasion of bladder cancer cells is regulated by p38MAPK-driven MK2 through stabilization of MMP-2/9 transcripts [58].

#### Role in prostate cancer

Transforming growth factor β (TGFβ) is an important regulator of cell adhesion and motility in a wide range of cell types including prostate and is shown to act in a cell-specific manner [64, 65]. Past studies have shown that TGFβ-mediated increase in cell invasion in human prostate cancer is dependent upon p38MAPK activation [66]. It has also been observed that both Smad3 and p38MAPK are integral for TGFβ-mediated cell adhesion in prostate cancer [66]. Recent studies have suggested that during colorectal cancer progression, TGFβ promotes tumor growth via its involvement in crosstalk with different pathways like p38MAPK and Wnt [67]. A study by Xu et al. demonstrated that in human prostate cancer both MK2 and Hsp27 are important for TGFβ-mediated up-regulation of MMP-2 activity and cell invasion which was inhibited by SB203580, a p38MAPK inhibitor [50]. This clearly indicated a direct role of p38MAPK signalling in prostate cancer through a channelized activation of p38MAPK, MK2 and Hsp27 (Fig. 4).

## MK2 and mRNA stability (interplay between MK2, RBPs and target RNAs)

### Adenine/uridine-rich elements (AREs)

Cytokines are essential for cell signaling to facilitate responses to various stimuli necessary for the maintenance of homeostasis and survival. Any malfunction in the cytokine signaling network has damaging effects on both the intra-cellular as well as the extracellular environments. An important process in cancer pathogenesis is cytokine and growth factor dysregulation which causes uncontrolled cell growth. Hence, tight regulation of the expression of cytokines at transcriptional and post-transcriptional levels is critical. AREs are conserved sequences located in the 3′-UTR of short-lived transcripts that codes for a multitude of proteins responsible for apoptosis, cellular activation, cytokine signaling, and growth. The stability of cytokine mRNAs has been shown to be altered due to the presence of AREs in their 3′-UTRs.

In 1986, conserved AREs were found in the 3′-UTR of genes encoding short-lived cytokines (granulocyte-macrophage colony-stimulating factor (GM-CSF) and TNFα) [68]. A lot of information about the role of AREs in the post-transcriptional regulation of many cytokines and growth factors is available [69]. AREs act as binding sites for RBPs that regulate mRNA half-life [70]. Most of the RBPs that bind to AREs target them to exosome thereby promoting rapid deadenylation and degradation of their substrate mRNAs (e.g., TTP, AUF1) [71]. Approximately 10–15% of all mRNAs comprising a functionally diverse pool responsible for cellular proliferation, development inflammatory and immune response, RNA metabolism, signaling, and transcription harbour AU-rich sequences [72, 73]. The recently constructed human ARE-containing mRNA database encompasses greater than 1000 transcripts [74]. Within the 3′-UTR, the presence of an ARE is a common link in many unstable mRNAs in mammals which is a part of the regulatory system responsible for the mRNA degradation or stabilization and is linked to interaction with RBPs [75]. The fate of ARE-containing mRNAs is determined by the integration of functionalities of multiple ARE-binding proteins/RBPs [76].

3′-UTR located AREs constitute cis-elements causing rapid degradation of transcripts encoding many cytokines, growth factors, and proto-oncogenes [77]. It has been shown previously and confirmed by findings in MK2^−/−^ mice that the p38MAPK/MK2 pathway facilitates transcript stability of mRNAs that harbour distinct AREs [8, 24, 78]. In comparison, the stability of ARE-deficient mRNAs was not affected [79]. The molecular mechanisms behind the control of ARE-mediated stabilization/decay involve the activity of proteins selectively interacting with ARE, but their mode of action is not well elucidated [80].

AREs comprises of many large clusters of overlapping AUUUA pentamers repeats and UUAUUUAUU nonamers that are specifically recognized by a variety of different ARE-binding proteins and found in transcripts encoding various cell cycle regulators (p16, p21, p27, cyclins, and Cdks), cytokines, epidermal growth factor (EGF), insulin-like growth factor (IGF), proto-oncogenes (c-fos, c-jun, c-myc), TGFβ, and vascular endothelial growth factor (VEGF) [77]. The list has considerably increased as a result of various genome sequencing programs [72]. Continuously active mRNA-decay mechanisms restrict the cytokine expression in resting cells. mRNA stability regulation through AREs is a post-transcriptional control mechanism which allows cells under varying environmental conditions to fine-tune the expression of important gene products (reviewed in [81]).

### RNA-binding proteins (RBPs)

RBPs are single or double-stranded RNA binding proteins present in cells which participate in the formation of ribonucleoprotein complexes and portray pivotal roles in processes such as cellular functions, transport, and localization. They are responsible for post-transcriptional control of RNAs, such as pre-mRNA splicing, and polyadenylation, as well as mRNA export, turnover, localization, and translation [82]. Apart from regulating mRNA decay, RBPs mediate other post-transcriptional processes like intracellular localization, pre-mRNA processing, translation, and transport (reviewed in [83]). Various reports have highlighted the function of multiple diverse classes of RBPs in the regulation of mRNA decay and stabilization (reviewed in [84]).

Studies have indicated the role of MK2 in the modification of the stability and translation of IL-6 and TNFα mRNA via activation of RBPs such as TTP, AUF1, and HuR (Table 1). These processes of complex post-transcriptional cytokine synthesis regulation *via* MK2-mediated RBPs phosphorylation have been discussed in some excellent reviews [85, 86]. A number of proteins having the potential to bind to ARE are known, among them TTP, and AUF1 stimulate target transcript decay by recruiting deadenylases and downstream degradation machinery [87]. By contrast, the embryonic lethal and abnormal vision (ELAV) family member HuR stabilizes its targets by competing with the destabilizing ARE-binding proteins for ARE occupancy (reviewed in [87, 88]). Induction of decay pathways for mRNA allows for the attenuation of cellular cytokines production through interactions with RBPs [89].

During inflammatory responses, cytokine mRNAs are stabilized via complex interactions with RBPs controlled by phosphorylation *via *multiple signaling pathways including the MAPKs. Activation of p38MAPK stabilizes the COX-2 transcripts *via* its effect on AUF-1, HuR, and TTP [90]. Substantial evidence has highlighted the relevance of mRNA stability in the regulation of genes [91]. mRNA fate is regulated by the complex interplay among the cis-acting sequences within mRNA and trans-acting nuclear and cytoplasmic factors [92]. The mammalian genome encodes approximately 1000 RBPs which portray important roles in mRNA stability, splicing, localization, nuclear export, and translation. RBPs physically interact with mRNA to exert their functionality in a highly sequence-specific manner. AREs are amongst the well-characterized regions that bind RBPs. Different RBPs have been discovered which function by stabilizing, destabilizing or influencing the translation of ARE-containing mRNAs (Table 1). A possible hypothesis for role of the p38MAPK cascade is that it stimulates the modification of RBPs by phosphorylation. RBPs are rightly called as the master regulators of transcript processing and translation with their expression often found to be aberrant in cancer [93]. In conjunction with much-studied transcription factors, RBPs have emerged as integral components in tumor development. RBPs along with their mRNAs targets form a complex network of post-transcriptional regulation of gene expression that plays a crucial role in tumorigenesis [94].

#### Tristetraprolin (TTP)

One of the substrates of MK2 is TTP [16, 95]. TTP is a critical anti-inflammatory RBP that presents transcripts to the decay machinery to enhance their mRNA decay [96]. Many essential proteins like cytokines are regulated by post-transcriptional TTP-mediated destabilizing mechanisms (reviewed in [97]). The role of TTP as a trans-acting anti-inflammatory RBP first came into light when TTP^−/−^ mouse showed overexpression of TNFα in macrophages and developed the pro-inflammatory phenotype [98]. TTP confers mRNA instability and degradation by binding the conserved ARE in the 3′-UTR of transcripts [99], which promotes the poly(A) tail shortening reported in GM-CSF and TNFα [100]. TTP shows very low constitutive levels and is an early response gene induced in phagocytes by LPS. It functions as a negative feedback on cytokine mRNAs; hence, mice lacking TTP tend to overproduce cytokines. Contradictorily, TTP expression is influenced by p38MAPK signaling [16]. TTP becomes hyperphosphorylated, with both p38MAPK and MK2 having been implicated in this process [16, 95].

The p38MAPK pathway regulates the mRNA expression, mRNA decay property and protein expression of TTP via MK2 [101]. TTP binds the TNFα ARE and destabilizes the mRNA [99]. Mice null for TTP develop an inflammatory syndrome because they overexpressed TNFα [98]. MK2 phosphorylates 14–3-3 binding sites at Ser52 and Ser178 in TTP [95], causing the protein to be sequestered away from TNFα mRNA and prevents it from recruiting a deadenylase to the bound transcript [102, 103]. These phosphorylations enable complex formation of TTP with multifunction adaptor 14–3-3 proteins resulting in ablation of its function as a transcript destabilizing protein [95], hence, allowing efficient translation *via* subcellular translocation of the mRNA [51, 104]. Literature suggests that MK2-mediated TTP phosphorylation increases the expression of TTP protein *via *cytoplasmic retention and exclusion from proteasomal degradation [105]. TTP dephosphorylation causes its movement from the cytoplasm into the nucleus and causes its degradation [101]. The deletion of AREs in the 3′-UTR of TNFα in mice leads to elevated TNFα production and inflammatory disorders [106].

Many studies have shown that TTP overexpression in vitro promoted the decay of mRNAs containing AU-rich sequences from TNFα [99]. In a p38MAPK-dependent manner, TTP directs mRNA stability of IL-6 [107]. The p38MAPK-MK2 axis is responsible for TTP being a mRNA destabilizing factor [100]. Moreover, in head and neck squamous cell carcinoma (HNSCC), TTP down-regulation enhances the stability of mRNAs, promotes IL-6 and VEGF secretion and significantly increases cellular invasion in cancers by increased secretion of IL-6 and MMP-2/9 [108, 109]. Given all these reports, TTP could be considered a therapeutic target as it can concurrently lead to down-regulation of multiple cytokines in HNSCC.

It was recently shown that TTP expression is inversely correlated with invasion in HNSCC [108]. In macrophages, TTP is inactivated by phosphorylation [51]. The mechanism by which TTP mediates invasion of HNSCC has been investigated, and it has been shown that the suppression or p38MAPK-mediated phosphorylation of TTP leads to the promotion of invasion due to enhanced secretion of IL-6 and MMP-2/9. TTP promotes mRNAs degradation by binding to AREs in the 3′-UTR [96, 107]. In macrophages, p38MAPK inactivates TTP *via* MK2-mediated phosphorylation at two serine sites [51, 103]. Typical targets of TTP are mRNAs regulating tumor growth such as TNFα, COX-2, VEGF, and IL-10 [110] (Table 1). It has been suggested that decreased TTP expression contributes to cancer-related processes, and reports show that TTP-mediated regulation of crucial cancer-related transcripts in breast cancer cell leads to suppression of their invasive potential [111].

#### Human antigen R (HuR)

HuR, one of the most prominent RBP, is intricately involved in tumorigenesis [112], with its overexpression been observed in a number of cancers including brain, breast, colon, gastric, lung, lymphomas, oral, ovarian, pancreatic, prostate, and skin cancers [113]. In normal cells, HuR is generally localized in the nucleus, but in transformed cells, it often translocates to the cytoplasm [114]. MK2 has been shown to induce the cytoplasmic accumulation of HuR [114]. MK2 has been shown to regulate intercellular adhesion molecule-1 (ICAM-1) and IL-8 expression in acute inflammatory response *via* HuR [115]. The sub-cellular localization of HuR is governed by post-translational modifications, and all the HuR modifying enzymes are implicated in cancer processes [116]. In the cytoplasm, HuR binds to AREs located in 3′-UTR of target mRNAs. HuR is most often functionally defined as a positive regulator of target mRNAs-stability and translation [112], which generally code for cyclins, favouring cell cycle progression and promoting proliferation of malignant cells [117]. In vivo models suggested a more diverse functional array with multiple complex side-effects [118]. Investigations suggested that elevated cytoplasmic localization of HuR corresponds to high-grade tumor thereby serving as a good prognostic indicator for poor clinical response in many cancers [119].

HuR targets mRNAs which encodes products promoting proliferation, increasing angiogenesis, inhibiting apoptosis, and facilitating invasion and metastasis, viz. COX-2, GM-CSF, IL-6, IL-8, inducible nitric oxide synthase (NOS), TGFβ, TNFα, VEGF, and others [120] (Table 1). IL-1β activates the MK2-HuR pathway which significantly enhances IL-6 mRNA stability and leads to the development of an inflammatory environment in glioblastoma [121]. HuR leads to the promotion of cancer cell survival through stabilization of transcripts encoding anti-apoptotic factors like B-cell lymphoma 2 (Bcl-2), p21, and Sirtuin 1 (SIRT1) [122]. The mechanism behind this feature of HuR is still unclear, but a few studies attribute this to the interplay among HuR and microRNAs [123]. HuR enhances the stability of a set of its target mRNAs by antagonizing their binding to RBPs or microRNAs that destabilizes them [124, 125]. Overexpression of HuR is found in HNSCC, and it leads to increases in the stability of COX2 and VEGF mRNAs [113]. In several cancers (including HNSCC) increased cytoplasmic HuR localization has been found, which contributes to increased COX-2 expression in metastasis and tumorigenesis [126].

#### AU-rich element RNA-binding protein 1 (AUF1)

AUF1 is a RBP that regulates the mRNA stability of proto-oncogenes, growth factors, cytokines, and cell cycle regulatory genes. AUF1 generally destabilizes transcripts and has been shown to control the stability and translation of GM-CSF, IL-6, TNF-α, VEGF and many other ARE-containing mRNAs [127] (Table 1). AUF1 has been reported to be present in a cytosolic fraction and its overexpression in animal models has been shown to be associated with decreased mRNA stability [128]. It has been shown that a p38MAPK-MK2-Hsp27 signaling axis promotes proteasomal degradation of AUF1, further leading to cytokine ARE-mRNAs stabilization [129].

## Correlation between MK2-mediated mRNA stabilization and tumorigenesis

An important determinant in modulating gene expression levels is the regulation of mRNA stability. Numerous studies in the past have demonstrated the importance of mRNA stability-mediated regulation in inflammation and cancer [9, 68, 89]. Modulation of the decay rate of various cytokines, proto-oncogenes, and growth factors, involves AREs in their 3′-UTRs [77]. RBPs tend to fine-tune cellular responses and directly mediate critical inflammatory signals responsible for disease pathogenesis by binding to AREs. It is quite evident that any aberrations in mRNA decay processes can lead to the over-production of certain gene-encoded products that can possibly lead to cancer. Post-transcriptional regulation of gene expression has been shown to be aberrant in tumors with over-expression of ARE-rich mRNAs been reported in multiple cancers [130]. RBPs like HuR have been shown to stabilize VEGF mRNA in various tumors [131]. AREs tend to play a huge role in the post-transcriptional regulation of certain genes involved in carcinogenesis [78]. Mechanistic insights on how AREs fine-tune mRNA stability reveal involvement of specific MK2-regulated RBPs [9, 132]. Past findings have implicated MK2 in mediating tumor invasion *via* regulating mRNA stability of MMP-2/9 in bladder cancer [58]. Hence, a better understanding of MK2-RBP mediated mechanisms will surely enable us to develop novel therapeutics in combating cancer progression [133].

## Therapeutic implications of MK2

### As therapeutic target?

MK2 modulates the stability and translation of inflammatory cytokines through phosphorylation of transactivating factors binding to their AREs [51]. Hence, MK2 inhibition could be a target for blocking the production of inflammatory mediators. Traditionally active site inhibitors of the kinases were employed for therapeutic purposes. But the major issue with this approach is that ATP competitive inhibitors of kinases have been known to be inherently cross-reactive, because of the homology shared by kinase active sites, hence, the development of specific active site kinase inhibitors is difficult.

A more viable approach in the development of selective kinase inhibitors is the search of agents that disrupt the docking among kinases, and their upstream and downstream signaling partners. MK2 docking domain comprising peptide is a potent inhibitor of p38MAPK-dependent phosphorylation of MK2. This might also perturb p38MAPK’s interaction with its upstream activators, like MKK3 [134]. Thus a more reasonable approach for inhibiting this pathway would be the development of inhibitors of the docking interactions between p38MAPK and its signaling partners [135].

### Inhibitors of MK2: Types, uses and history

Systemic side-effects of p38MAPK inhibitors such as cardiac toxicity, hepatotoxicity and central nervous system (CNS) disorders have been among foremost hurdles against the developed inhibitors to transform into a successful drug. This was the main reason behind their failure in phase III clinical trials [136]. To overcome the issue and for effective inhibition of p38MAPK signalling pathway, researchers prompted toward numerous downstream targets of the pathway such as MK2 [10].

Currently, MK2 is widely considered as a novel disease-modifying anti-rheumatic drug (DMARD) ligand and a promising possible alternative to p38MAPK for the treatment of various inflammatory diseases. Study on the involvement of MK2 in inflammation associated disorders suggested that health of p38^−/−^ mice suffering from embryonic lethality and loss of fertility was more severely affected as compared to the MK2^−/−^ mice [137]. Furthermore, low levels of inflammatory cytokines have been observed in the brain and serum of MK2^−/−^ mice in addition with limited or no symptoms in arthritis and lung sensitization models [10]. Along with it, neuro-protective effect has been observed after MK2 depletion indicated towards the association of neuro-inflammation with neurodegenerative disease such as parkinson’s disease, multiple sclerosis and even alzheimer’s disease. So it has been suggested that this linkage could be directly associated with modulation of MK2 activity [10].

Past studies had indicated that targeting MK2 to block its downstream events could be equivalent to direct inhibition of upstream p38α (responsible for MK2 activation) of the p38MAPK pathway, with the additional advantage of lacking any p38-dependent side effects [138, 139]. This is the reason that MK2 is currently being considered as a more promising target. The inhibitors of the MK2 activity could serve as potential therapeutic agents in the treatment of various inflammation and neuro-inflammation associated diseases. The active involvement of MK2 with Hsp27 may also be used to reduce remodelling and migration of cancer cells and metastasis through its abrogation. Furthermore, considering the capability of MK2 to modulate a cell cycle checkpoint, inhibitors of MK2 are also considered as effective tools to evade DNA repair mechanism induced by chemotherapy and thus resulting in increased sensitivity of tumor cells to chemotherapy [25, 58, 60].

Nearly all of the revealed MK2 inhibitors belong to the type I class of inhibitors (ATP competitive MK2 inhibitors (which binds to the ATP binding site of the kinase) and therefore compete with intra-cellular ATP molecules to block p38MAPK-mediated phosphorylation and activation of the kinase. Several compounds with in vivo efficacy against MK2 have been already reported by other researchers also [140]. After discerning various compounds with minimal to modest in vitro activity towards MK2 [141], researchers have made significant improvements in efficacy and safety as compared to compounds generated earlier. However, low biochemical efficiency (BE) value (generally expressed as the ratio between Ki - the binding affinity of inhibitor molecule to the target protein and its effective concentration 50 (EC50-cellular activity of the inhibitor) has been one of the major drawbacks of the MK2 inhibitors discovered so far.

### Void and lacunas in the area of MK2 inhibitor research

Various studies on the mechanism of action of total marketed drugs demonstrate that around two-thirds of them have BE values higher than 0.4 [142]. A study by Swinney et al. [142] reported that BE value higher than 0.4 is an attribute of many approved drugs. If we conclude strictly, the EC50 values for any successful drug should not be more than 2.5-folds higher than its Ki values. Studies indicated that cellular efficacy reports for MK2 inhibitors in a diseased condition are quite inadequate in public domain, and indicated BE values of the tested inhibitors are far below the 0.4 threshold, suggesting the unlikeness of available MK2 inhibitors to become successful drug candidates [140]. Retaining in mind the fact that high concentrations of inhibitor compounds are required to ascertain good cellular efficacy in diseased conditions, their cytotoxicity, non-specificity and side-effects could be aggravated, thus increasing the probability of attrition. Conversely, compounds not competing with intracellular ATP could remain active at comparatively lower concentrations and have a greater probability to be optimized to become a drug. Inopportunely, the currently available uncompetitive and non-ATP competitive MK2 inhibitor compounds do not provide any experimental support to this hypothesis, thus, opening the door of possibilities for experimental validation of already available non-competitive MK2 inhibitors.

The higher affinity of inactive MK2 towards intracellular ATP has been anticipated as the major determinant of lowering the BE values for potential MK2 inhibitors. Consequently, researchers have screened their known inhibitors among the pool of compounds that bind the inactive form of the kinase, have a lesser competition with the high intracellular ATP concentration, and, accordingly, are required at low concentrations to give cellular effects in diseased conditions. By looking into all these factors, the importance of MK2 in modulating inflammatory conditions, cell cycle process, cytoskeleton remodelling and cell motility, non-ATP-competitive and allosteric inhibitors of MK2 are under continuous investigation as negative regulators or modulators of the p38MAPK/MK2 signalling pathway in various disorders [10].

### Current insights into MK2 inhibitors

#### ATP competitive inhibitors

MK2 has been hypothesized as a potent druggable target in inflammatory disorders. The release of 3D structure of MK2 in complex with ADP or other small molecule inhibitors drove the discovery of numerous small molecule ATP-competitive inhibitors (Table 2). Conversely, blocking the MK2 with its ATP binding site in competitive mode gave rise to two important challenging issues. Firstly, the similarity of the ATP-binding site of MK2 with other kinases (MK3, MK5, etc.), interferes with the selectivity of inhibitors. Secondly, low BE value of the ATP-competitive inhibitors either due to high affinity of ATP for its binding site on kinase. Finally, solubility in suitable agents and permeability profiles of inhibitors appropriate for in vivo administration have been very difficult tasks to be addressed [10, 141].

**Table 2:**
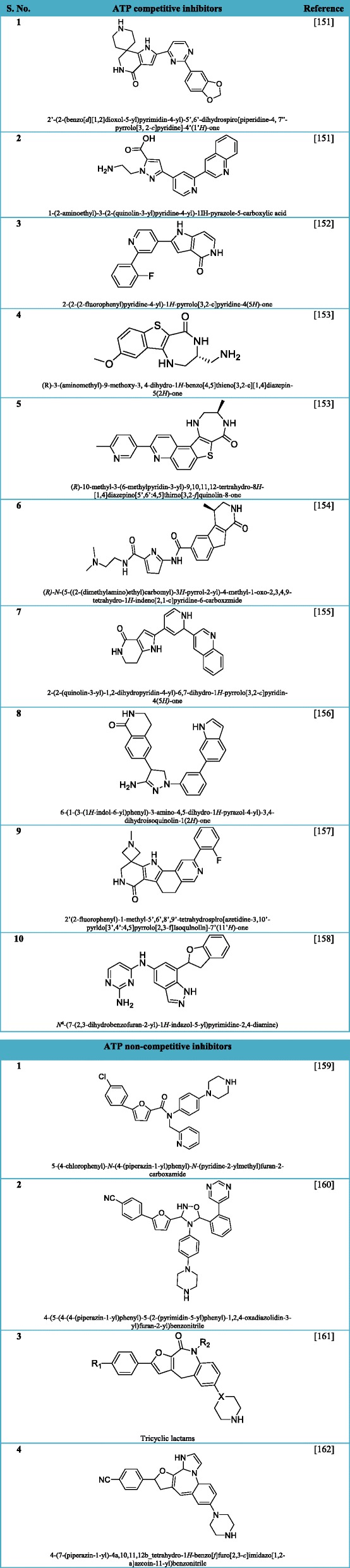
List of potent ATP competitive and non-competitive inhibitors of MK2 [151–162]

The list provided here is not comprehensive and is made using the most frequently cited inhibitors currently under investigation to design MK2-targeting therapeutics

#### ATP non-competitive inhibitors

In the recent years, due to the inefficiencies associated with ATP-competitive inhibitors, promising inhibitors with non-ATP competitive and ATP-uncompetitive mechanism of action have been identified (Table 2). These compounds have the distinctiveness to interact with a binding site in kinase which is different from that of ATP, thus avoiding issues like selectivity with other kinases and low BE value. Additional advantage associated with them is effectiveness at low concentration. By definition, non-competitive inhibitors are not required to contend with the high ATP concentrations in cells and with high affinity of ATP for the inactive and active forms of MK2, effective lower concentrations of them promises less pronounced side-effects also. Mechanism of action of inhibitor dissimilar to ATP-competitiveness could enhance BE value of potential inhibitors and have better possibilities to be developed as an effective drug candidate against MK2. Thus, inhibitory efficacy of a non-ATP competitive inhibitor is expected to be higher than ATP-competitive inhibitors. Additionally, they could exert higher kinase selectivity profile as a consequence of the fact that they do not bind to similar ATP binding sites among related kinases [10].

Studies have shown that a good BE value enables the efficacy of a drug at lower concentrations with an increase in the therapeutic index, there is a minimum probability of success in clinical studies in case of ATP-competitive MK2 inhibitors. Mourey et al. [140] demonstrated in vivo efficacy of a selective ATP-competitive MK2 inhibitor PF-3644022 despite its biochemical inefficiency (BE 50.03). This inhibitor has been reported to reduce TNFα production in inflammation mice models. Various non-ATP-competitive inhibitors have been reported by Merck [143], and it would be of a topic of great interest to see the progression of this class of compounds in in vivo and clinical studies further (Table 2). As of now, the outcomes of MK2 inhibition can only be assumed and solely depend on the analysis of the efficacy of inhibitors of p38MAPK that target MK2 activation. Along these lines, Watterson et al. [144] have recently demonstrated that the anti-neuroinflammatory efficacy of blood-brain-barrier-permeable p38MAPK inhibitors in the animal model of Alzheimer’s disease correlates with the inhibition of MK2 activity. Recently, CDD-450, also called ATI-450 was developed as an unique inhibitor which possesses the property of selectively blocking p38MAPK-mediated MK2 activation while sparing other p38α substrates. ATI-450 has an efficacy similar to global p38α inhibitors and inhibits IL-6, IL-1β, and TNF-α production thereby decreasing inflammation in preclinical models [145].

## Conclusions

MK2 activation generates a plethora of different biological effects targeting diverse cellular processes like cell-cycle progression, cytoskeletal architecture, mRNA stability, and protein translation *via* regulating the activation and deactivation cycles of RBPs [146, 147]. Improved understanding of MK2’s role in tumor progression could provide new insight into the enigma behind the post-transcriptional gene regulation in tumorigenesis. The complex mechanisms of post-transcriptional of cytokine regulation via MK2-mediated phosphorylation of RBPs play a pivotal role in tumorigenesis [85, 86].

Inhibition of the p38MAPK/MK2 pathway by blocking p38MAPK failed, as none of the inhibitors were found successful in the clinical trials due to the unwanted side effects [10]. Hence, in recent times, MK2 was preferred as a potential candidate for targeted therapies as a p38MAPK alternate to minimize the systemic undesired effects associated with the majority of p38MAPK inhibitors. MK2 remains a promising therapeutic target given the importance of the p38/MK2 pathway in processes like cell-cycle, inflammation, and metastasis.

DNA-damage due to chemotherapeutic agents could be repaired by cancer cells by arresting cell cycle progression and escaping apoptosis. It has been shown that MK2 activity is essential for G2/M arrest, hence; it gives an exciting prospective for the utility of MK2 inhibitors as chemo-sensitizers. Importantly, MK2-depleted mice are viable [47], in contrast to Chk1 and p38MAPK^−/−^ mice [148], suggesting that MK2 inhibition could target cancer cells the same way as Chk1 and p38MAPK inhibitors but with fewer side-effects. Latest reports of MK2 inhibition decreasing production of inflammatory cytokines and subsequently leading to reduced tumor volumes potentiates its use in therapeutics [149, 150].

Pathological roles of MK2 in several diseases has led to a renewed interest in developing drug-like MK2 inhibitors despite the difficulties encountered in this process. The identification of MK2 inhibitors with suitable pharmacodynamics and pharmacokinetics is an attractive question for medicinal chemists [10]. Scientific advances in the area of molecular oncology have opened novel research directions. Nowadays, numerous research endeavours have been concentrated towards developing targeted therapies and unveiling novel molecular markers which could be utilized in predictions of treatment outcome or personalized therapies. It is quite evident that further unraveling the molecular tumorigenesis enigma will surely pave the way forward for novel therapeutics and personalized treatment regimens for the patients.

## Abbreviations

3′-UTR: 3′-untranslated region
AOM/DSS: Azoxymethane/Dextran sodium sulphate
AREs: Adenine/uridine-rich elements
AUF1: AU-rich element RNA-binding protein 1
BCL-2: B-cell lymphoma 2
BE: Biochemical efficiency
CDC25: Cell division cycle 25
CNV: Copy number variation
COX-2: Cyclooxygenase-2
CREB: Cyclic AMP-responsive element-binding protein
DMARD: Disease-modifying anti-rheumatic drug
DMBA: 7,12-dimethylbenz[*a*]anthracene
EC50: Effective concentration 50
EGF: Epidermal growth factor
ELAV: Embryonic lethal and abnormal vision
ERK: Extracellular signal-regulated kinase
F-actin: Filamentous actin
GM-CSF: Granulocyte-macrophage colony-stimulating factor
HDM2: Human homolog of mouse double minute 2
HNSCC: Head and neck squamous cell carcinoma
HSP27: Heat shock protein
HuR: Human antigen R
IECs: Intestinal epithelial cells
IFNγ: Interferon-γ
IGF: Insulin-like growth factor
IL: Interleukin
LPS: Lipopolysaccharide
MAP2K: MAPK kinase
MAP3K: MAPK kinase kinase
MAPKAPK2 or MK2: Mitogen-activated protein kinase-activated protein kinase 2
MDM2: Mouse double minute 2 homolog
MK2^−/−^: MK2 knockout
MMP: Matrix metalloproteinase
NES: Nuclear export signal
NLS: Nuclear localization signal
NOS: Nitric oxide synthase
NPC: Nasopharyngeal cancer
NSCLC: Non-small cell lung cancer
p38MAPK: p38 mitogen-activated protein kinase
PKB: Protein kinase B
PLK1: Polo-like kinase 1
RA: Rheumatoid arthritis
RBPs: RNA-binding proteins
SDS-PAGE: Sodium dodecyl sulphate-polyacrylamide gel electrophoresis
SIRT1: Sirtuin 1
TGFβ: Transforming growth factor β
TIMP: Tissue inhibitor of metalloproteinases
TNFα: Tumor necrosis factor
TSC2: Tuberin
TTP: Tristetraprolin
UTR: Untranslated region
UV: Ultraviolet
VEGF: Vascular endothelial growth factor
